# The Esthetics of Surgery

**Published:** 1874-01

**Authors:** Ben. H. Pratt


### THE BISTOURY.

Vol. IX. ELMIRA, N. Y., JANUARY, 1874. No. 4.

### Contributions.

### THE ESTHETICS OF SURGERY.

BEN. H. PRATT, A. M.

Surgery, as a fine art, has never been sufficiently exemplified. At least, we have never met with any just tribute to this science for its refining element—for its power of developing taste—for its ability to call the practitioner from the material into the ethereal—for its genuine soul-elevating, mind-magnifying, humanitarian characteristics.

That it possesses all of the above excellences, and even more than these, no true observer of its practical workings—no deep thoughted student of its sublime effects can deny.

Without further preface, and to illustrate in what manner this esthetical development takes place, let us suppose a case. In surgery, medicine and law, we have nothing if no case. Let us suppose a "capital operation"—that is, a great operation—for while we have the privilege of choosing an imaginary case, let us take the best we can get—a case of ovariotomy.

Ovariotomy, may be, is a bigger word than the non-professional reader may be able to stagger under without assistance from a dictionary; and, as a dictionary is always a bother to the lazy reader, and we don't flatter ourselves that any busy man will stop to read the "long article" of even so considerate a publication as the Bistoury, we will "rise to explain."

Ovariotomy is the term used by the surgeon to designate the operation of removing an ovarian tumor, or, a tumor attached to the ovaries of some unfortunate human being. The ovaries are—well, no matter what they are, only they are inside of the abdomen of all females, away down in the lowest portion of the abdominal cavity, and sometimes—not often—these ovaries possess a tumor. It is a very serious matter, this tumor business, when it is connected with the ovaries, and we hope none of our readers will ever be afflicted with one. But some people will have them, and do, and that's why we are dwelling upon this point. Nobody knows why a tumor comes upon the ovaries, any more than they know why a boil comes upon the nose, or a carbuncle comes upon the back of the neck. They come, but unlike a boil or a carbuncle, they don't grow and burst and get well. They grow, and if let alone, will burst, but the possessor don't live long enough afterwards to tell how she felt when it burst. That's why we perform ovariotomy—or the removing of the tumor before it gets ripe and bursts into the abdomen and drowns the patient. For these tumors are very big, sometimes, and not unfrequently hold several pails of a thickish liquid which the Doctors call "serum"--people generally call it *matter*. Another bad thing about ovarian tumors, is that you don't know when they come. They don't produce any sensation, but just come, and grow, and grow, and grow, until by and by you know they're there from the bigness of yourself. This is the objectionable feature of this form of disease, and a terrible blow to the possessor of an ovarian tumor it is, when her Doctor tells her what the matter is. But nothing can be done about it at this stage of the malady. It can't be reduced by medicine—it won't be starved either. It grows on anything. If you don't eat, for fear of feeding the tumor, it will feed on the body, and thrive all the same. By and by it becomes very inconvenient, besides disfiguring the person. The pain is inconsiderable, but the mortification and dread are terrible, and this is the chief cause of bodily emaciation no doubt. Oftentimes the tumor grows to be heavier than the person who carries it. We recall a case in which the individual, tumor included, weighed 150 pounds," and the actual weight of the sac, or bag, and the liquid it contained, was 85 pounds by actual weight after removal.

Now, if you've got any idea of an ovarian tumor from the foregoing, we're about ready to show how surgery, as a fine art, developes the esthetical portion of man's nature.

A surgeon, sitting in his easy office chair, reading up some delightful description of some splendid operation, by some eminent brother, in some titled hospital or other, in some big city, in which is glowingly detailed how some poor devil was run over by the cars and all jumbled up into a mess of raw human meat, and how, after successfully lopping off his legs and arms—amputation they call it, as it sounds more refined—and after sewing him up here and there, and straightening him with bandage and compress and splint, were repaid by the knowledge that his life could have been saved if some clumsy doctor, who was left in charge of the patient for a few hours, hadn't let the man die from absolute ignorance of the simplest remedies for coma, or sinking from shock ; while, as we said, he is enjoying this bit of a professional treat, his morning mail is brought, and he begins to tear off the ends of the envelopes and hastily scans the contents of each. They're a queer lot, these Doctor's letters, full of odd questions, and serious questions, and funny questions, and a mixture of terror and nonsense, and entreaty—bad spelling and worse writing—details of tediousness, and the prolix complainings of diseased minds arising from bodies enfeebled by chronic maladies of one sort and another—a queer lot surely. But now the surgeon opens a letter that strikes him instantly as something worth while. It may run like this :

J. Scalpel Cleynkutt, M. D.

DEAR SIR:—

My wife has been getting very large in her bowels lately. Our doctor says it is a "varying tumer" and he can't do anything with it. He told me to write to you and you would know right off what to do. It began to show about last July, and has kept a growing ever since. Our doctor says you can take it out, and I want you to come and see her and talk it up with me. If you can cure her I'll make it worth your while to undertake it.—Waiting for a quick reply, I am

Yours Truly,

JOHN SMITH.

While reading this very short letter, the surgeon's eyes have twinkled several times. Upon its perusal he pokes the letter in his pocket and goes right home to tell his wife all about it. Then he dances a professional jig to work off the surplus joy that letter has brought him—then hunts up all his friends and tells them about it—tells them what a glorious time he's going to have—tells just how he's going to work it—who's going to help him—when he's going to see the case—tells it all with such a glee, and such a glorification of his art that you feel how wrapped up he is in his profession. His soul fairly dilates with the inspiration that bit of a letter has brought him. His powers are enlarged thereby. He don't dwell upon the difficulty of the work before him, nor upon the disagreeable features of the operation, nor upon the probabilities of failure, nor upon the responsibility he is about to assume. His ideas float far above these material matters. He roams at large through the ideal realms of his chosen science. He reflects how he can add another star to the galaxy of the art restorative—meditates what new process he can bring to bear upon his pending case—what new alleviative influence he can submit to the surgical world that will cause his brethren to rise up and call him benefactor—dwells indeed among the etherealities, and is nourished by the esthetics of his case.

He visits his patient—arranges the preliminaries—fixes the time—settles upon the fee—goes home—examines his glittering case of instruments—takes each one out lovingly and inspects it thoroughly—touches up the edge of this and the point of that to artistic keenness—chamois them all into brilliancy—makes up bandages—prepares his sutures—living all the while in an ecstasy of thought, and counting the days, nay, even the hours intervening between him and the appointed time. Can there be anything that develops the esthetic more completely than a mental process like this ?

The hour arrives. The patient is ready—the surgeon is prepared. There's no nonsense about this. Success, life and health are in one scale—failure, death and mourning are in the other. The feather that is to cause either end to kick the beam is in the surgeon's hand. The patient has been warned of the possible event—death. She is reconciled. She has been told of the probable issue—successful removal of the difficulty, and restoration to health. She is hopeful. The anæsthetic is administered—conciousness has fled—the surgeon is in bliss, with his favorite scalpel poised over his inanimate patient. Patient, indeed, to those on-looking, but to him who has chosen to abide the issue—who has staked his reputation, skill, knowledge, and a fame that is dearer than life to him, upon this operation—the motionless being lying there is a grand and glorious Opportunity only. She is his Divinity—whether of good or ill, remains to fate, but whether of good or ill, no less his Divinity. Within that prostrate form rests that, which, if overcome, sets science to writing another line to add to individual fame ; sets the humanities to writing another line to add to merciful surgery. All is ready. Dread seizes the spectators, yet it is a dread mingled with a fascination. The scene is a study for the pencil of the artist—a theme for the pen of the writer—a chapter for the scientist—an essay for the humanitarian. A human being, one made after the image and likeness of God, possessing a soul that must either soon put off its mortality at the bidding of disease, or go again among the living, a vital monument to the achievements of scientific art; part forever from near and dear to the realms of the vast unknown, or abide for indefinite time amid the dear ones and the loved ones ; become the mourned of the stricken, and the remembered of the bereaved, or the loved presence of the household and the cherished saved. The surgeon stands, scalpel in hand, an emblem of a divinity—a personification of Salvator or of Mors—one who may save or kill, as perchance his superiors, the Triune Fates, may elect. The grouped friends, relatives, sympathizers—the waiting attendants and assistants—the jury of Medicine called in to witness a resurrection or dissolution—the significant silence, and the growing awe, lest, mayhap, Death is even now brandishing his spear, and angel attendants are waiting to bear hence a released soul—strange tableau, and varied emotions these, and strangely they affect.

The skilled eye of the master, however, sees but his art. Bodily, he is there—in spirit, he is threading the paths of esthetic ecstasy, and gathering from either hand therealong, delights not earth-given. That instant of waiting is to him an age of realized bliss—a cycle of unalloyed enchantment. We cannot follow him thereto. It were almost a sacrilege so to attempt.

But ere we are aware the knife has been skillfully applied—the parts are opened—the tumor is revealed—the attachments carefully released—the parting of the normal and the abnormal are speedily effected—the great pedicle is severed—the thing is removed and borne away—the bowels of the living are visible—the stomach, the liver, the net-work of blood vessels and the pulsations thereof, all are revealed—the hemorrhage is arrested—the parts carefully treated—the incision closed and secured by sutures—the bandages applied—the result is awaited—the merciful anæsthetic has served its purpose—life returns—the eyes open upon gathered friends—consciousness resumes sway—a smile lights up the countenance of the saved one—she is informed of what has transpired—is assured of her safety. Care and judicious nursing, with absolute quiet and proper nourishment—these supplement the grand act in the drama of a regenerated body, and the completed work challenges a reverence for the man whose science has achieved a temporary victory over death, that is near of kin to adoration.

These are the tangible—the material, the sensible evidences of surgery ; but who can fathom the immaterial—the etheral—the occult influence thus wrought within the soul of him who has effected this almost miracle ?

He who can tell this knows how replete with the esthetical is the surgeon's work—knows the by-paths of his reverie—is well acquainted with the dreamy labyrinths of that romanceful realm, wherein those esthetical flowers are culled which, though unseen, do none the less bind the brow of the chiefest of humanitarians, the surgeon devoted to his art.

